# Lineage-Specific Evolved MicroRNAs Regulating *NB-LRR* Defense Genes in *Triticeae*

**DOI:** 10.3390/ijms20133128

**Published:** 2019-06-26

**Authors:** Rongzhi Zhang, Shujuan Zhang, Weiwei Hao, Guoqi Song, Yulian Li, Wei Li, Jie Gao, Yongsheng Zheng, Genying Li

**Affiliations:** 1 Institute of Crop Science, Shandong Academy of Agricultural Sciences; Key Laboratory of Wheat Biology and Genetic Improvement on North Yellow and Huai River Valley, Ministry of Agriculture; National Engineering Laboratory for Wheat and Maize, Jinan 250100, China; 2 State Key Laboratory of Plant Cell and Chromosome Engineering, Institute of Genetics and Developmental Biology, Chinese Academy of Sciences, Beijing 100101, China

**Keywords:** microRNAs, disease resistance genes, miRNA target genes, miRNA expression, miRNA evolution

## Abstract

Disease resistance genes encoding proteins with nucleotide binding sites and Leucine-Rich Repeat (NB-LRR) domains include many members involved in the effector-triggered immunity pathway in plants. The transcript levels of these defense genes are negatively regulated by diverse microRNAs (miRNAs) in angiosperms and gymnosperms. In wheat, using small RNA expression datasets and degradome datasets, we identified five miRNA families targeting *NB-LRR* defense genes in monocots, some of which arose in the *Triticeae* species era. These miRNAs regulate different types of *NB-LRR* genes, most of them with coil-coiled domains, and trigger the generation of secondary small interfering RNAs (siRNA) as a phased pattern in the target site regions. In addition to acting in response to biotic stresses, they are also responsive to abiotic stresses such as heat, drought, salt, and light stress. Their copy number and expression variation in *Triticeae* suggest a rapid birth and death frequency. Altogether, non-conserved miRNAs as conserved transcriptional regulators in gymnosperms and angiosperms regulating the disease resistance genes displayed quick plasticity including the variations of sequences, gene copy number, functions, and expression level, which accompanied with *NB-LRR* genes may be tune-regulated to plants in natural environments with various biotic and abiotic stresses.

## 1. Introduction

The competition between plants and pathogens has never disappeared, and sometimes, it becomes very intense. During evolution, plants evolved multiple adaption mechanisms for biotic stresses. The important defense mechanisms include PAMP-Triggered Immunity (PTI) triggered by Pathogen-Associated Molecular Patterns (PAMP) as a preliminary defense [1] and Effector-Triggered Immunity (ETI) as a secondary defense [2]. In the ETI pathway, plants developed Nucleotide-Binding site and Leucine-Rich Repeat (NB-LRR) proteins to recognize effector and to trigger the ETI response [2], which can cause programmed cell death and lead to hypersensitive response (HR) [3]. *NB-LRRs* with a large gene family are an important class of disease resistance genes. Of the total coding genes, 1.19–3.48% were defined as *NB-LRR* genes in plants [4]. According to their N-terminal features in plants, NB-LRR proteins can be functionally classified into two classes based on the presence of terminal Toll/Interleukin-1 Receptor (TIR) or Coiled-Coil (CC) domains [5]. TIR and CC domains always play a very important role in transmitting signals to cellular targets for effector actions or downstream signaling components [6]. Although *NB-LRR* genes have been demonstrated as ancient and conserved genes in plants [7], comparative genomics analysis has shown great structural diversity. For example, CC domains were dominantly present in eudicots and monocots, while TIR domains were nearly absent in monocots [7]. Approximately 85% of verified disease resistance genes contained CNL (*CC*-NB-LRR) or TNL (TIR-NB-LRR) domains [4]. In addition, the wild resources have abundant disease resistance genes. For example, in *Triticum*, the defense genes *Sr31* and *Sr50* with CNL [8] from cereal rye (*Secale cereale*) can confer the resistance to stem rust disease caused by *Puccinia graminis f. sp. tritici* (*Pgt*). *Sr35* gene with CNL from *Triticum monococcum* confers the resistance to Ug99 Stem Rust Race Group [9,10]. 

Most of the genes involved in the disease defense pathways always have very rapid evolutionary rates in the coevolution process with pathogens [11]. The high expression of the *NB-LRR* class genes will do damage to the growth and development of plants [12]. Thus, plants evolved a regulatory mechanism in the posttranscription level to balance the expression level of disease resistance genes [13].

MicroRNAs (miRNAs), as a class of small noncoding RNAs, function in posttranscriptional gene regulation. Small miRNAs play important roles in a variety of biological processes, such as development, hormone responses, and stress adaptation [14,15,16,17]. Recently, many studies have confirmed the role of plant miRNAs in response to pathogen challenge. In the last two decades, many experimental efforts have aimed to demonstrate how miRNAs shield plants from pathogen attack. MiRNAs respond to virus, bacteria, and fungi by negatively regulating mRNAs, which mainly includes two classes in both PTI and ETI [18,19,20,21,22,23,24,25,26,27]. One type of miRNAs negatively regulates targets to obtain basal resistance in the PTI pathway. For example, miR393 in *Arabidopsis* can be elicited by Flagelin 22 (flg22). Repressing auxin signaling through miR393 overexpression increases bacterial resistance to virulent Pto DC3000 by negatively targeting *F-box auxin receptors* transcripts *Transport Inhibitor Response 1* (*TIR1*) [18]. The miR169/*Nuclear transcription Factor Y*, *Alpha* (*NFYA*) module in *Arabidopsis* influences *Ralstonia solanacearum* pathogenicity [19] and in rice against the blast fungus *Magnaporthe oryzae* [20]. The *Arabidopsis* miR396/*GRF* module mediates innate immunity against *P. cucumerina* infection without growth costs [22]. MiR444/*OsMADS* directly monitors *RNA-dependent RNA polymerase 1* (*OsRDR1*) transcription involved in the rice antiviral pathway [23]. Increasing rice miR319 expression level or decreasing the expression of its target *TCP21* can facilitate rice ragged stunt virus infection via the jasmonic acid pathway [24]. Overexpression of osa-miR171b contributed to less susceptibility to a rice stripe virus infection by regulating SCARECROW-LIKE6 (*OsSCL6*) [25]. Interference with miR773 activity upregulates the target gene *METHYLTRANSFERASE 2* (*MET2*) in *Arabidopsis* and elevates the resistance to infection by fungal pathogens [26]. 

In addition to basal defense, miRNAs can also directly regulate disease resistance genes in the ETI pathway. These disease resistance genes have proteins that include NB, LRR, TIR, and CC domains, most of which are mediated by the generation of 21-nt phased siRNAs (phasiRNAs) [28]. The suppression of regulatory networks by miRNAs and disease resistance genes may play an important role in plant–microbe interactions via small RNA silencing mechanisms [28]. Disease resistance genes provide defense against pathogen stresses by multiple duplication and diversification of the gene dosage expression level, while miRNAs minimize the cost of gene copies by suppressing their expression [29]. One miRNA sometimes can regulate dozens to hundreds of disease resistance genes posttranscriptionally by targeting similar motifs [13], which make it more economical to balance the benefits and costs of these copies in the genome.

The regulation of the *TIR-NB-LRR* and *CC-NB-LRR* gene classes by miRNAs has mostly been characterized in dicots. In reported posttranscriptional regulation networks, miRNAs can trigger 21-nt phasiRNAs generation from *NB-LRR* transcripts, which are further processed by RNA-Dependent RNA polymerase 6 (RDR6) and DICER-LIKE 4 (DCL4) [30]. For example, *Brassica* miR1885 was validated to be induced by a *Turnip mosaic virus* (*TuMV*) infection, which induced the cleavage of *TIR-NB-LRR* transcripts [31]. By cleaving *TIR-NB-LRR* immune receptors in *Tobacco*, both nta-miR6019 and nta-miR6020 contributed to the resistance to *Tobacco mosaic virus* (*TMV*) [32,33]. Sl-miR482f and sl-miR5300 controlled NB domain-containing proteins at the translation level and are involved in plant immunity in tomato through posttranscriptional regulation [34]. In *Arabidopsis*, miR472 influenced disease resistance genes mediated by the RDR6 silencing pathway [35]. In *Medicago*, miR2109, miR482/miR2118, and miR1507 were found to regulate the *NB-LRR* gene family [13]. MiR1507, miR1510, miR2109, and miR482 also targeted the *NB-LRR* gene class with TIR or CC domains in legumes, which were proposed to function in the modulation of defense response during rhizobium colonization [30,36]. Additionally, miR482/miR2118, miR951, miR946, miR950, miR1311, miR1312, miR3697, miR3701, and miR3709, etc. have also been shown to mediate the generation of phasiRNAs by targeting the *NB-LRR* genes in *Norway Spruce* [37]. However, very few miRNAs were identified as the regulator of disease resistance genes in monocots, which may be due to the structure variation of *NB-LRR* genes such as the absence of TIR domain, and the neofunctionalization of disease resistance-associated miRNAs such as miR2118 in monocots. Until now, only one miRNA family was validated as the regulator of *NB-LRR* genes. MiR2009 was first predicted to target *Mla* alleles in wheat [38]. In barley, the miR9863 family homologous to tae-miR2009 was confirmed to trigger responses to *Mla*-mediated immune signaling, which is fine-tuned at a later stage of MLA activity to avoid overloading of immune responses in barley cells [39].

Increasing numbers of small RNAs from high-throughput sequencing are available in the public database, including various developmental and stress samples. Multiple efforts have been made to identify the differential expression of miRNAs in response to biotic and abiotic stresses [13,30,31,32,33,34,35,36,37]. In grasses, aside from miR9863 which is described above, few miRNAs have been validated as major regulators of disease resistance genes. Despite these efforts, our knowledge about the role of miRNAs in response to disease resistance genes in grasses is still limited. Here, taking wheat as an example, we identified five disease resistance-associated miRNAs that target disease resistance genes with NB, LRR, or CC domains. They also initiated the generation of secondary phasiRNAs in the target transcripts. These miRNAs had an independent evolutionary pathway compared with eudicots and had high diversification with absence or expansion variation in *Triticeae*. These periodic nascent miRNAs by regulating the *NB-LRR* genes may maintain the potential fitness or growth costs in *Triticeae* species at the posttranscriptional level. 

## 2. Results

### 2.1. Identification of Disease Resistance-Associated MiRNAs in Wheat

**Figure 1:**
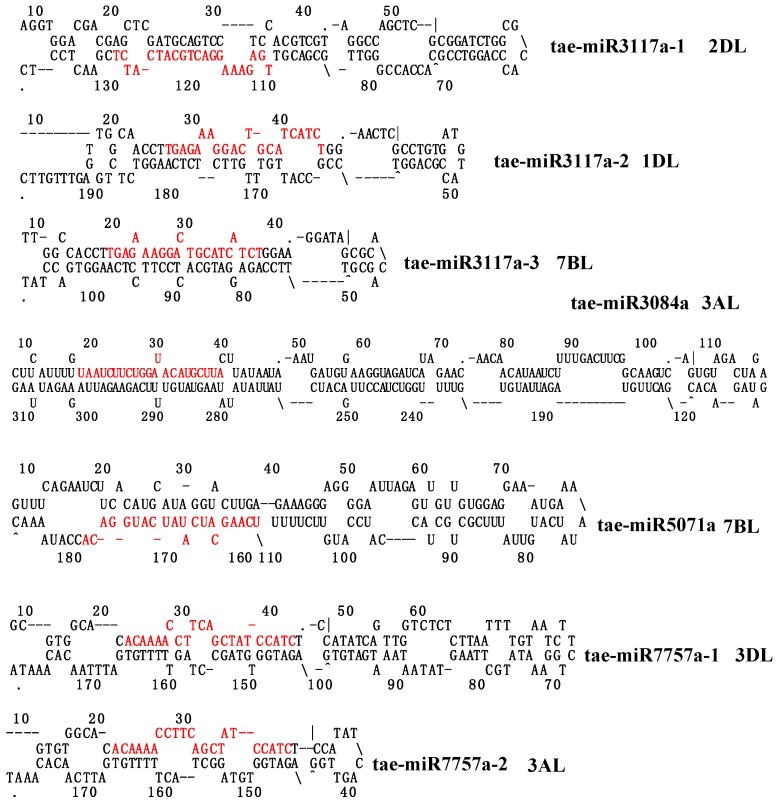
The precursors of *TAE-MIR3117a*, *TAE-MIR3084a*, *TAE-MIR5071a*, and *TAE-MIR7757a* disease resistance-associated miRNAs. The red letters represent the mature miRNA sequences.

Comparing the miRNAs from the miRbase [40], PNRD [41], and PmiRExat [42] websites to the target sequences in the CDS (Coding Sequence) and UTR (Untranslated Region) of genes with the Targetfinder program in wheat [43], five miRNA families in wheat were characterized to target disease resistance genes with typical NB and LRR domains. Mi3117, miR3084, miR5071, miR7757, and miR9863 had two, two, two, two, and four members, respectively (Figure 1 and Table 1). These were tae-miR3117a, tae-miR3117b, tae-miR3084a, tae-miR5071a, tae-miR7757a, and tae-miR9863a. For tae-miR3117b, there is only one mismatch with tae-miR3117a. The lengths of tae-miR3084a, tae-miR5071a, and tae-miR9863a were 22 nt, while tae-miR3117a-b and tae-miR5071 were 21 nt in length (Table 1).

In barley, the hvu-miR9863 had been characterized to regulate *NB-LRR* genes by triggering the biogenesis of 21-nt phased siRNAs [39]. In wheat, the tae-miR2009 family, which was identified by Wei et al., [38], is homologous to hvu-miR9863 and also targets *mildew resistance locus* (*Mla1*). Here, we designated tae-miR2009 as tae-miR9863a in the next section.

Next, we mapped the miRNA sequences to the wheat genome using the BLAST program with word size parameter—W 7 and an equal length and 100% identity between the query and subject sequences [44]. Tae-miR3117b had 252 loci mainly located in subgenome A (90%, 228/252), while tae-miR3117a only had eight loci located on chromosomes 1AS, 1DL, 2AL, 2DL, 3AS, 3AL, 7AL, and 7BL (Table 1 and Supplementary Table S1). For tae-miR3084a, only one copy was located in the genome on chromosome 3AL. For tae-miR5071a, there were six loci on 3AL, 3B, 5BL, 6BS, 7BL, and an unknown chromosome. For tae-miR7757a, there were three loci on the 3AL, 3B, and 3DL chromosomes. After mapping these miRNAs and truncating the flanking sequences in the wheat genome, the miRNA precursors were predicted with the mfold website (http://unafold.rna.albany.edu/) [45]. After filtering the precursors with a dG less than −30, *TAE-MIR3117A* displayed three loci on chromosomes 1DL, 2DL, and 7BL; *TAE-MIR3084A* had one locus on chromosome 3al; *TAE-MIR5071A* has one locus on chromosome 7BL; and *TAE-MIR7757A* had two loci on chromosomes 3AL and 3DL (Figure 1). The lengths of the precursors varied from 130 to 310 nts. The multiple loci of miRNAs indicated the special genome expansion in *Triticeae*.

### 2.2. The Expression and Stress-Response of Disease Resistance-Associated MiRNAs

**Figure 2:**
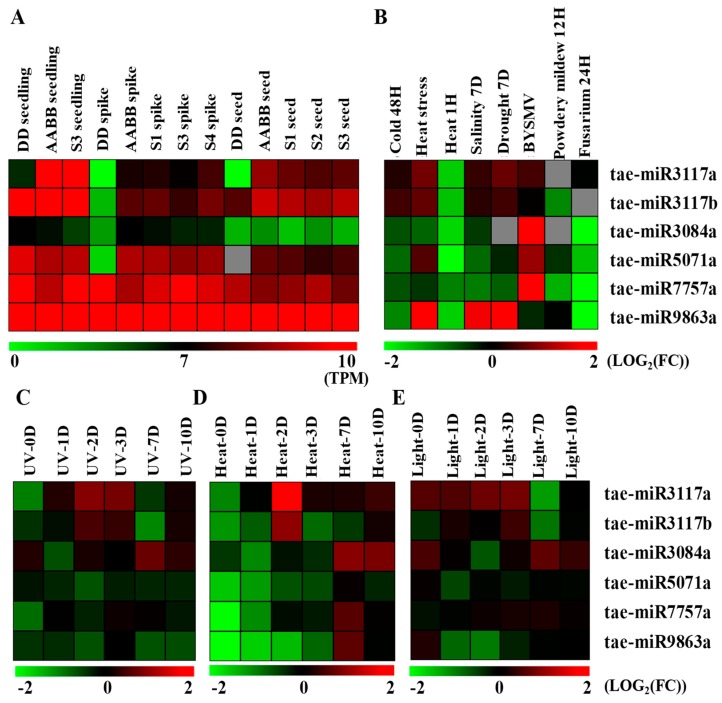
(**A**) The expression profiles of disease resistance-associated miRNAs in *Triticum* including diploid (AA and DD), tetraploid (AABB) and hexaploid (AABBDD including S1, S2, S3, and S4) wheat. (**B–E**) The expression responses of disease resistance-associated miRNAs under biotic stresses including *BYSMV*, powdery mildew, and *Fusarium* and abiotic stresses including cold, heat, salinity, drought, UV, and light. The gray color is the absence of an expression value.

Using the small RNA sequencing libraries from the GEO database, we detect the expression of the disease resistance-associated miRNAs (Supplementary Table S2). To unify the standards, we used the transcripts per million (TPM) value to standardize all of the samples (Supplementary Tables S3 and S4). In the tetraploid (AABB, *Triticum turgidum*), diploid (DD, *Aegilops tauschii*), and nascent allohexaploid (AABBDD including S1, S2, S3, and S4 wheat generations), tae-miR9863a had higher expression levels in the young seedling, spike, and seed developmental stage; tae-miR7757a had moderate expression levels; and tae-miR3804a had relatively low expression levels. For example, tae-miR9863a had a TPM value higher than 27,000 in *Aegilops tauschii* seedling. Tae-miR7757a had a TPM value ranging from 100 to 1700, and tae-miR3084a had a TPM value ranging from 3 to 40 (Figure 2A and Supplementary Table S3). Tae-miR3117a-b displayed a seedling-specific expression in tetraploid and hexaploid wheat, with TPM values greater than 20,000 in the S3 seedling, but was absent or had very low expression levels in the seedling, spike, and seed of *Aegilops tauschii*. Tae-miR5071a also displayed moderate expression levels but not in the spike and seed developmental phases in *Aegilops tauschii*. Therefore, the disease resistance-associated miRNAs may regulate the abundance of disease resistance genes with spatial and temporal specificity in diploid, tetraploid, and hexaploid wheat (Figure 2A and Supplementary Table S3).

Biotic and abiotic signal pathways may share multiple nodes, and their outputs may have significant functional overlap. For example, overexpression of the *TIR-NB-LRR* gene *VaRGA1* of grapevine could enhance disease resistance and can improve the drought and salt tolerance in *Nicotiana benthamiana* [46]. Also, enhancing the expression of the *CC-NBS-LRR* gene *ADR1* could improve the drought tolerance via the salicylic acid and abscisic acid pathways [47]. Disease resistance genes are induced when the biotic or abiotic environment is perturbed [48]. Thus, to detect the response of the disease resistance-associated miRNAs to biotic and abiotic stresses, we detected the miRNA expression variations in the small RNA libraries including biotic stresses such as powdery mildew, *barley yellow striate mosaic virus* (*BYSMV*), and *Fusarium* infection and abiotic stresses such as thermal stress (high and low temperatures), salt stress, drought stress, light, and UV stresses (Figure 2 and Supplementary Table S4).

Here, we defined the values (log_2_(fold change)) of gene expression level between the mock/ck and the stressed samples greater than 1 or less than −1 as upregulated or downregulated, respectively. Among biotic stresses, 24 h after *Fusarium* infection, tae-miR3084a, miR5071a, miR7757a, and miR9863a were downregulated while tae-miR3117a-b showed no changes in the expression level. For a *powdery mildew* infection, only tae-miR7757a was downregulated while other miRNAs did not show any changes in their expression levels. However, for the *BYSMV* infection, the disease resistance associated miRNAs were upregulated for tae-miR3084a, tae-miR5071a, and tae-miR7757a (Figure 2B and Supplementary Table S4A).

Among the abiotic stresses, for heat stress, three independent research groups sequenced small RNAs in response to high temperature or heat stress. All the results demonstrated that the disease resistance-associated miRNAs were downregulated at the beginning of heat stress at 0 h [49], 1 h [50], and 1 day [49] after heat stress (Figure 2B,D and Supplementary Table S4B,C). Two days after heat [49], tae-miR3117a-b was upregulated while tae-miR9863a was downregulated (Figure 2D and Supplementary Table S4B). For the mixed samples treated with 30 min, 2 h, 4 h, and 8 h of heat stress [51], tae-miR9863a was upregulated (Figure 2B and Supplementary Table S4C). At 3, 7, and 10 days of heat treatment, there were no obvious changes in the expression of the disease resistance-associated miRNAs (Figure 2D and Supplementary Table S4B) [49]. Generally, downregulated disease resistance-associated miRNAs may respond to heat stress through an initial transmission signal in order to accumulate *NB-LRR* genes, which can allow plants to initiate the response mechanisms. Thus, these miRNAs are shown to be downregulated and then to gradually become upregulated to repress the overexpressed *NB-LRR* transcripts in order to make them reach a normal expression tendency. For cold stress [52], no miRNAs were shown to be up-/downregulated (Figure 2B). For salinity stress [51] and drought stress [51], only tae-miR9863a was upregulated (Figure 2B and Supplementary Table S4C). In response to light stress [49], only tae-miR3117a-b was downregulated at light-7d, and there was no apparent expression changes under UV stress [49] (Figure 2C,E and Supplementary Table S4B). Thus, all the disease resistance-associated miRNAs showed responses to heat stress, though only a few of them responded to salt, drought, and light stress. This demonstrated that the disease resistance-associated miRNAs may be involved in the response to the various environments and pathogens.

### 2.3. The Target Prediction of Disease Resistance-Associated MiRNAs

Using the Targetfinder program [43], 34, 21, 57, 60, and 33 genes were respectively identified as targets of tae-miR3117a-b, tae-miR3084a, tae-miR5071a, tae-miR7757a, and tae-miR9863a [43] in wheat CDS and UTR sequences. By analyzing the target domains using the Hmmscan [53] and MARCOIL programs [54], we found that 91.18% (31 of 34), 90.48% (19 of 21), 98.25% (56 of 57), 91.67% (55 of 60), and 93.94% (31 of 34) targets of the tae-miR3117a-b, tae-miR3084a, tae-miR5071a, tae-miR7757a, and tae-miR9863a targets, respectively, contained NB, LRR, or CC domains (Supplementary Table S5). Seven, one, thirty, and sixteen genes in the NB domain regions and eight, zero, sixteen, and seven genes in the NB-CC domain regions contained the target sites of tae-miR3084a, tae-miR5071a, tae-miR7757a, and tae-miR9863a, respectively. In contrast, none of tae-miR3117a-b target genes had target sites in their NB, LRR, or CC disease resistance domains and only one site was located in the 5′UTR region (Supplementary Table S5). Tae-miR3117a-b may have special regulation sites in the *NB-LRR* transcripts compared with other four miRNAs.

### 2.4. The Potential Regulation of MiRNAs Interacting with Their Targets

**Figure 3:**
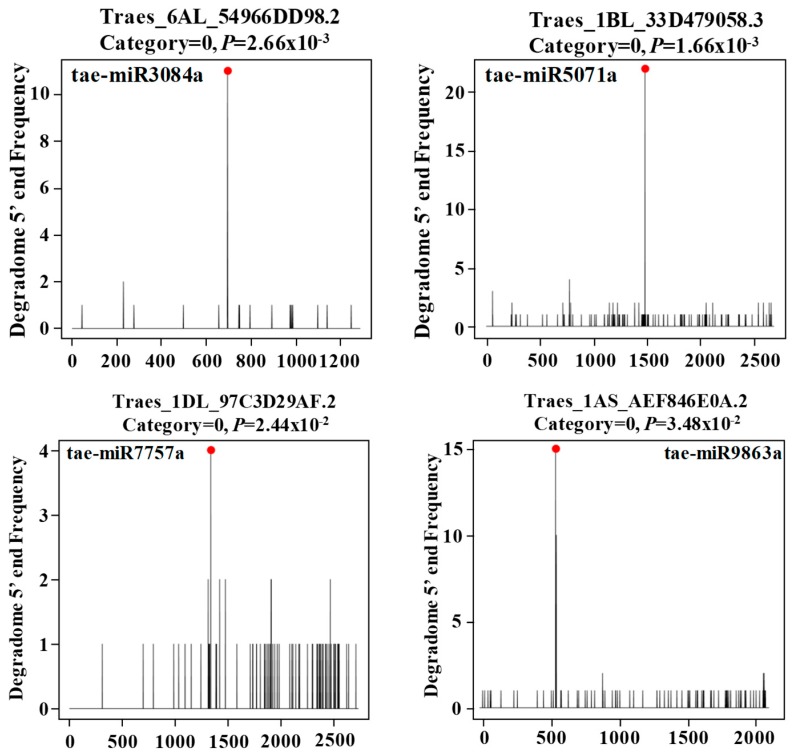
Target plots (T-plots) of miRNAs characterized in the degradome datasets: The abundance of signature tags was plotted along the indicated transcripts. The red dots indicate the predicted cleavage sites on the x-axis, and the black lines indicate the signatures produced by miRNA-directed cleavage.

In barley, it has been demonstrated that tae-miR9863a guides the cleavage of *Mla1* disease resistance gene transcripts [39]. To validate whether these miRNAs can indeed cleave the predicted targets, we downloaded the degradome sequences from the GEO datasets for the young spike tissues under cold stress by Song et al. [52] and Tang et al. [55]. Using the CleaveLand program [56], we classified the cleavage of the target-miRNA as 0, 1, 2, 3, and 4 categories according to the abundance of reads along the whole transcripts (Table 2 and Supplementary Table S6). Totals of five, twenty-two, three, and sixteen miRNA-target cleavage sites were detected for miR3084, miR5071, miR7757, and miR9863, respectively. One, two, three, and three genes with category 0 for tae-miR3084a; seven (5(0), 1(2), and 1(3)), nine (7(0), 1(2), and 1(4)), fourteen (11(0), 1(1), and 2(2)), and fourteen (11(0), 1(1), 1(2), and 1(4)) for tae-miR5071a (Number in the parentheses indicate the category of the miRNA-target cleavage); one (1(0)), zero, two (2(0)), and one (1(0)) gene for tae-miR7757a; and ten (9(0);1(1)), eight (7(0);1(2)), eight (5(0);1(1);1(3)), and seven (6(0);1(3)) genes for tae-miR9863a were confirmed to be cleaved with *P*-values less than 0.05 using the degradome sequences from the control and cold-stressed libraries by Song et al. [52] and Tang et al. [55] (Supplementary Table S6 and Figure 3). In total, tae-miR3084a, tae-miR5071a, tae-miR7757a, and tae-miR9863a were verified to cleave 5, 22, 3, and 16 gene targets, respectively, using the degradome sequences in at least one library. However, for tae-miR3117a-b, none of targets were validated to be cleaved targets, as tae-miR3117a-b may be specifically expressed in seedling leaves. This indicated that these miRNAs may interact with their targets via a cleavage mechanism.

### 2.5. The Conservation of the Target Site Regions

**Figure 4:**
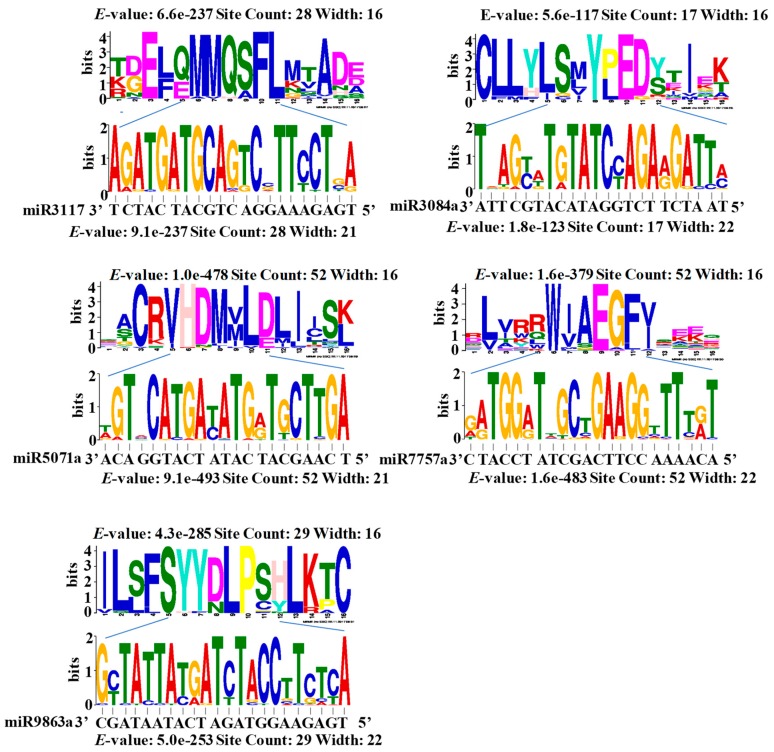
Motif diagram in the target sites of the disease resistance-associated miRNA: The logo diagrams show the alignment of the RNA and protein sequences in the target site and their flanking regions. The site count entries describe the number of sequences used in each alignment.

To understand the conservation of the target regions regulated by miRNAs, we first truncated the target site sequences complementary to the miRNAs and then used the MEME program to detect whether the sequences were consistent. The motif logo showed the high identity of the miRNA-target interaction regions with an *E*-value less than 10^−200^. At the 9th, 10th, or 11th site from the miRNA 5′-site, no sequence variation was observed (Figure 4). We then curtailed the upstream and downstream protein sequences flanking the target site regions with more than four amino acid residues and used the MEME program to detect the consistency of the sequences. The logo diagram showed that the sequences from the targeted regions were much more consistent than their flanking regions, with *E*-values less than 10^−100^ (Figure 4). During soybean domestication, the genetic density of the flanking region was much higher than the miRNA binding sites in miRNA target genes [57]. Combined with these results, it deduced that the sequences of target site regions may have a lower evolution rate than their flanking regions.

### 2.6. The Generation of the Secondary SiRNAs in the Target Transcripts

**Figure 5:**
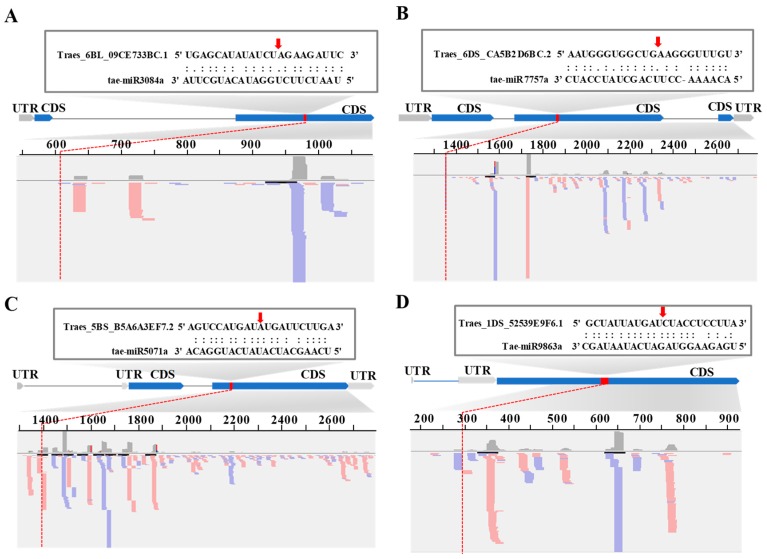
Phased siRNA distributions of the (**A**) tae-miR3084a, (**B**) tae-miR5071a, (**C**) tae-miR7757a, and (**D**) tae-miR9863a target transcripts: The red or blue color represents the positive or negative strand orientation of the small RNAs in the bottom panel. The red arrow represents the target transcript candidate cleavage site.

MicroRNAs that were either 21 nt or 22 nt in length can always activate the generation of secondary siRNAs in a phased pattern in their target mRNAs, which are called phasiRNAs [30]. In plants, various genes, such as *MYB*, *PPR*, and *NB-LRR*, can produce an enormous number of phasiRNAs in a head-to-tail arrangement triggered by the 21-nt miRNAs such as miR390 [58] and miR161 [59] and by the 22-nt miRNAs such as miR482/miR2118 [60,61,62], miR1509 [13], and miR2275 [61,62]. Here, using the Short Stack package [63], we aligned the small RNA datasets downloaded from the GEO database to the target transcripts of miRNAs. Nineteen phased clusters were identified with a phased score greater than 15 in the 17 small RNA libraries. Except for tae-miR3117a-b, the targets of tae-miR3084a, tae-miR5071a, tae-miR7757a, and tae-miR9863a could generate 21-nt phasiRNAs in the small RNA libraries at different developmental stages such as seedling and spike or under different stresses such as light stress (Figure 5 and Supplementary Table S7). These data demonstrated additional evidence for phasiRNAs generation in *NB-LRR defense responsive* genes triggered by miRNAs.

### 2.7. The Response to Various Pathogens for the Target Genes

**Figure 6:**
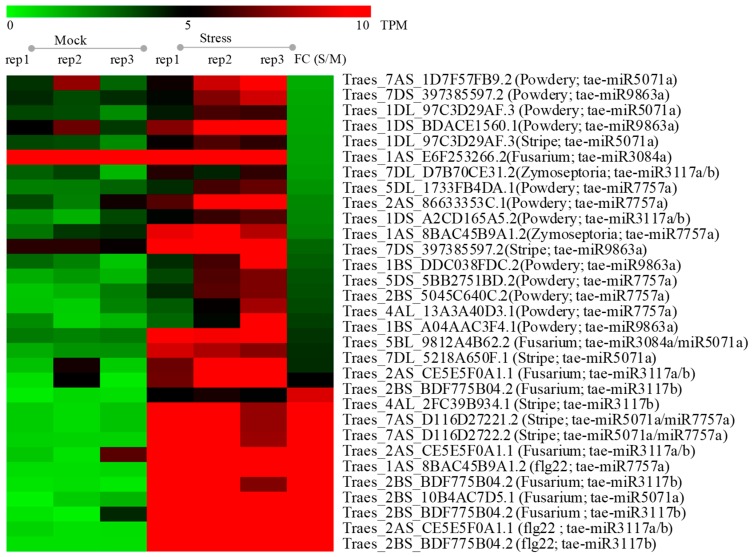
The heat map of target genes between the samples of mock and pathogen stress with three replicates. FC (S/M): Fold change (Stresses (rep1 + rep2 + rep3)/Mocks (rep1 + rep2 + rep3)). Fusarium, *Fusarium graminearum*; Powdery, *Powdery mildew*; Stripe, *Stripe rust*; Zymoseptoria, *Zymoseptoria tritici*.

Disease resistance genes have been reported to be involved in the defense of pathogens such as *Mla-like* (Accession number: AY487917.1), *Mla16-1* (Accession number: ACZ65488.1), *RPP13* (Accession number: EMS57774.1) *Sr35* (Accession number: AGP75918.1), and *Yr10* (Accession number: AAG42168.1). In this study, we identified 163 *NB-LRR* genes targeted by miRNAs in total. To understand the response of these targets in the infection of pathogens, we used the transcriptome datasets to investigate the variation of gene expression level with the fold change more than 1.5 times (Figure 6 and Supplementary Table S9). There are one, two, one, and three genes upregulated after 6 h, 48 h, 50 h, and 4 days inoculated with *Fusarium graminearum*, respectively. Five and one genes were upregulated after being infected by stripe rust pathogen for 1 and 3 days, respectively. There are twelve genes involving in the response to the infection of the *Powdery mildew* pathogen for 3 days. One and one gene were triggered to be upregulated after the *Zymoseptoria tritici* inoculation for 1 and 9 days, respectively. Also, three genes were upregulated after treatment with flg22 (500 nM). Targets of Tae-miR3117/tae-miR5071/tae-miR7757/tae-miR9863 can respond to the diseases of both stripe rust and *Powdery mildew* pathogens (Figure 6 and Supplementary Table S9); targets of tae-miR3117/tae-miR7757 can respond against the infections of both *Zymoseptoria tritici* and flg22 (Figure 6 and Supplementary Table S9); and targets of tae-miR3117/tae-miR3084/tae-miR5071 can be involved in the stress of *Fusarium graminearum*. Both targets of tae-miR3117 and tae-miR7757 could be responsive to the infection of Fungi and Bacteria (Figure 6 and Supplementary Table S9). Thus, these targets of disease resistance-associated miRNAs may play a role in the various disease-related stresses caused by the pathogens.

### 2.8. The Phylogenetic Relationship of the Target Genes

**Figure 7:**
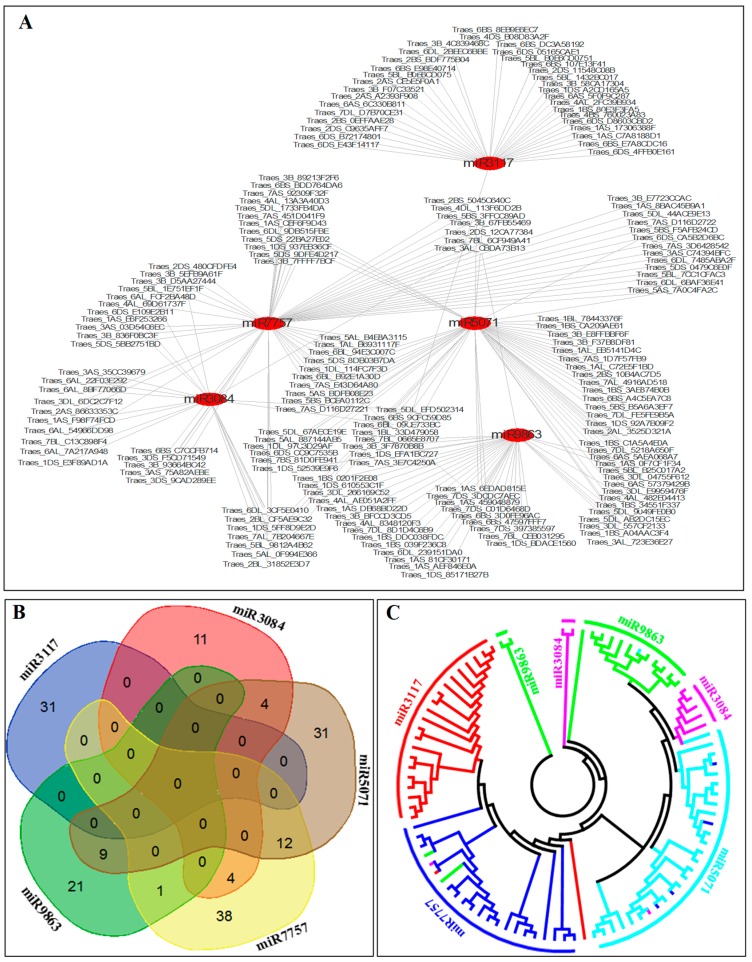
(**A**) The posttranscriptional regulation network between the miRNAs and their targets. The (**B**) Venn diagram and (**C**) phylogenetic relationship tree for the target genes of disease resistance-associated miRNA.

To understand the regulation relationship between miRNAs and their targets, we examined miRNA-target regulatory networks. As shown in Figure 7A, one miRNA typically regulated a cluster of *NB-LRR* defense-responsive genes. In Figure 7B, the Venn diagram showed that there were no targets shared by all five miRNAs. There were no targets shared between tae-miR3117a-b and the other miRNAs (Figure 7A,B and Supplementary Table S8). Only a few of the targets overlapped among the tae-miR3084a, tae-miR5071a, tae-miR7757a, and tae-miR9863a miRNAs. Nine genes were targeted by tae-miR9863a and tae-miR5071a, and one target overlapped between tae-miR9863a and tae-miR7757a. Twelve targets were shared between tae-miR7757a and tae-miR5071a, four targets overlapped between tae-miR7757a and tae-miR3084a, and four targets overlapped between tae-miR5071a and tae-miR3084a (Figure 7A,B and Supplementary Table S8). These results demonstrated that one disease resistance gene may be regulated by two or three miRNAs.

To further investigate the evolutionary relationship of the targets, we created a phylogenetic tree using MEGA software [64]. It is clear that the targets were classified as nine different groups based on the five miRNAs (Figure 7C). Two, one, one, two, and three miRNA-target groups were categorized for the targets of tae-miR3117a-b, tae-miR7757a, tae-miR5071a, tae-miR3084a, and tae-miR9863a, respectively. The targets of each miRNA could be grouped well except for a few of the targets. Two, one, and one targets of tae-miR9863a, tae-miR3084a, and tae-miR3117a-b, respectively, were clustered in the tae-miR7757a group, while four and one targets of tae-miR7757a and tae-miR3084a, respectively, were included in the tae-miR5071a group (Figure 7A,B). The less overlapped targets of the five disease resistance-associated miRNAs may indicate the specific regulation in each miRNA-target motif region.

### 2.9. The Evolution of Disease Resistance-Associated MiRNAs in Grasses

**Figure 8:**
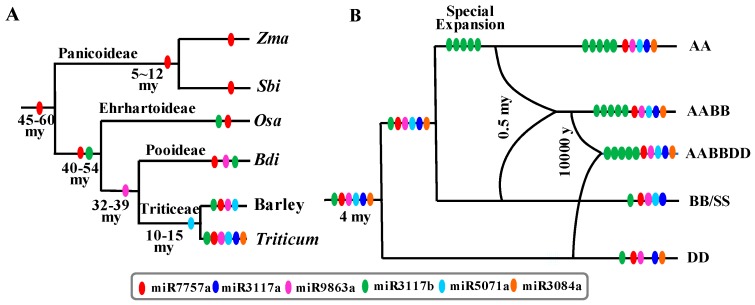
The evolutionary path of the disease resistance-associated miRNAs in (**A**) grasses and (**B**) *Triticum* species.

To investigate the evolution of disease resistance-associated miRNAs in grasses, we mapped the mature miRNA sequences to grass genomes, including *Panicoideae* (maize and sorghum), *Ehrhartoideae* (rice), *Pooideae* (*Brachypodium*), and *Triticeae* (barley, wheat, and their wild-relative species). Then, based on the conserved and species-specific miRNAs among grasses and *Triticeae*, we deduced the evolution history of the five miRNA families. According to the divergence time among species, we created the phylogenetic trees in grasses [65,66,67] and *Triticum* species [68]. As shown in Figure 8A, miR7757a was present in all the species. MiR3117b was conserved in rice, *Brachypodium*, barley, and wheat; miR9863a was conserved in *Brachypodium*, barley, and wheat; miR5071a was conserved in barley and wheat; and miR3117a and miR3084a were conserved in only *Triticum*. Thus, we can assume that tae-miR7757a emerged before the diversity of grasses approximately 45-60 million years ago (mya). MiR3117b appeared before the diversity of *Ehrhartoideae* and *Pooideae* approximately 40–54 mya, and miR9863a developed before the diversity of *Brachypodium* and *Triticeae* approximately 32–39 mya. MiR5071a was present before the diversity of barley and *Triticum* species approximately 10–15 mya. In contrast, miR3117a and miR3084a only appeared in *Triticum* species approximately 4 mya.

In *Triticum* species, we mapped the mature miRNA sequences to their genome, including AA (*Triticum urartu*), A^m^A^m^ (*Triticum monococcum*), SS (S^sh^S^sh^, *Aegilops sharonensis*, and S^s^S^s^, *Aegilops speltoides*), DD (*Aegilops tauschii*), AABB (*Triticum durum*), and AABBDD (*Triticum aestivum*). As shown in Figure 8B, tae-miR3117b has expanded in these species, particularly in AA, AABB, and AABBDD, all of which are contained in the AA genome but not in the BB and DD genomes. Thus, we can propose that tae-miR3117b had a special expansion in the AA genome approximately 0.5 mya before the emergence of the tetraploid AABB genome. Tae-miR3084a was lost from the SS genome. Since only a single copy of tae-miR3084a is present in the hexaploid and tetraploid wheat, we deduced that the loss of tae-miR3084a occurred before the tetraploid wheat generation approximately 0.5 mya. For tae-miR5071a, it disappeared in the DD genome, whereas present in the hexaploidy AABBDD genome, it was only distributed in the AA and BB subgenomes. Thus, it is possible that tae-miR5071a was lost approximately 10,000 years before the generation of the hexaploid wheat. Above all, these data indicated the dynamic rapid birth and death of disease resistance-associated miRNAs on a very short historical timescale.

## 3. Discussion

Flowering plants are derived from a common ancestor approximately 135 to 250 mya. During the evolution process, plants passed through countless encounters with pathogens. The pathogens evolved various tactics to adapt to the surrounding environment via changeable genomes. Thus, plants had to constantly develop strategies to defend against pathogenic virulence. The most important mechanism was to increase the copy number and diversity of disease resistance genes at the dosage and structure levels. The increase in gene expression level could be regulated when no pathogenic infection was present for cost benefit. Thus, plants developed another mechanism, i.e., transcript regulatory miRNAs, to control gene expression. To affect multiple disease resistance gene variations, miRNAs had to be fickle for survival with rapid plasticity including gene copy number variation, expression level variation, and function variation, etc.

### 3.1. The Disease Resistance-Associated MiRNAs and their Targets may be Involved in Several Disease Resistance Responses

Until now, several publications had demonstrated that some miRNAs could respond to the infection of pathogens such as virus [30,32,33] and fungus [39] by regulation of the *NB-LRR* transcripts in plants. Here, using the small RNA transcriptome datasets, we also detected that some miRNAs could respond to the biotic stresses. Tae-miR3084, tae-miR5071, tae-miR7757, and tae-miR9863 in wheat were downregulated after 24 h of a *Fusarium* infection, and miR7757 was also downregulated after a powdery mildew infection (Figure 2B). In addition, the targets of disease resistance-associated miRNAs could also respond to the disease infection by upregulating the gene expression level. Targets of these miRNAs could be involved in several disease resistance responses. For example, the targets of tae-miR3117 and miR5071 could be upregulated two to 44 times after infection by stripe rust, *Powdery mildew*, and *Fusarium graminearum.* Moreover, both tae-miR3117 and tae-miR7757 can also be upregulated after an infection of *Zymoseptoria tritici* and flg22 (Supplementary Table S9). Thus, miRNA-target regulatory networks may be involved in various disease resistance responses in grasses and may reduce the costs of disease resistance genes in plants when there is no pathogenic infection.

### 3.2. The Frequent Birth/Death of Disease Resistance-Associated MiRNAs with Gene Copy Number Variation between Eudicots and Dicots

**Figure 9:**
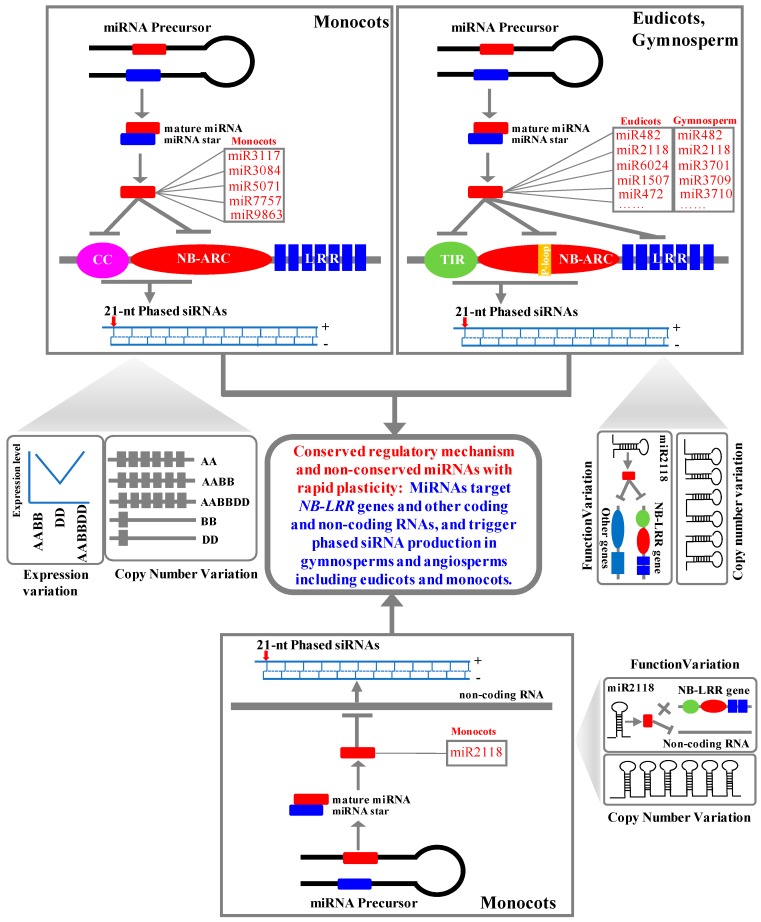
The conservation mechanism of miRNAs regulating the *NB-LRR* genes in plants in the top panel, their rapid plasticity including expression level in the left middle panel, their copy number in the right middle panel, and their function in the bottom panel.

For the development-associated miRNAs, such as miR156, miR172, miR319, miR168 and so on, it is clear that they are very ancient between eudicots and dicots. For the disease resistance-associated miRNAs, eudicot plants evolved a batch of miRNAs, including miR482/miR2118, miR472, miR1510, miR1503, miR6019, miR6020, and so on, to target *NB-LRR* genes (the top panel of Figure 9), which are described in the Introduction section. MiR482/miR2118 are ancient miRNAs in seed plants like gymnosperms (the top panel of Figure 9) such as *Norway Spruce* [37] and angiosperms such as *Arabidopsis*, *Brassicaceae*, *legume* species, etc. Most of miRNAs targeting *NB-LRR* genes are lineage-specific, such as the *Brassicaceae*-specific miRNAs including miR472, miR825, and miR1885; *Fabaceae*-specific miRNAs including miR1507, miR1510, miR2089, and miR5213; and *Solanaceae*-specific miRNAs including miR6024, miR6025, miR6026, miR6027, and miR5300 [28]. The lineage-specific evolution feature of the disease resistance-associated miRNAs was both in monocots and eudicots.

In grasses, miR9863 is characterized as inhibiting immune response signaling by targeting the *Mla immune receptors* in barley [39]. Except for miR9863, no other microRNAs that regulate *NB-LRR* genes were reported to be involved in immune response. Here, we found five miRNA families that regulate *NB-LRR* gene families in grasses. These miRNAs still behaved in the presence/absence of frequent variation, as shown in Figure 8 and the middle pannel of Figure 9. MiR7757 is the only ancient miRNA concurrent in *Panicoideae*, *Ehrhartoideae*, and *Pooideae*. MiR3117 was conserved in *Ehrhartoideae* and *Pooideae*. MiR9863 was conserved in *Pooideae*. MiR5071 was generated in *Triticeae*, and miR3084 evolved in the *Triticum* time. In grasses, five miRNA families were found to target *NB-LRR* families. Thus, we can propose that grasses gave birth to one novel miRNA to regulate the disease resistance genes approximately 8–9 mya.

On the large timescale, birth/death, and functional diversity of miRNAs are very clearly observed in plants including eudicots and dicots. There are no conserved miRNAs to regulate the *NB-LRR* genes. Disease resistance-associated miRNAs regulated the *NB-LRR* genes with CC domains in monocots, while in eudicots, they target the *NB-LRR* genes with TIR and CC domain [28] (the top panel of Figure 9). On a small timescale, taking *Triticum* as an example, we investigated the dynamic presence/absence of disease resistance-associated miRNAs. The expansion and deletion events, such as the special expansion of miR3117b in the AA genome, the deletion of miR3084 from the BB genome, and the deletion of miR5071 from the DD genome, demonstrated the high birth/death frequency over a 4-million-year selection process (Figure 8). In addition, miR482/miR2118 in litchi underwent a copy number expansion in tandem repeat [69] (the middle panel of Figure 9). It demonstrated that the mechanism with which miRNAs target *NB-LRR* genes and trigger phasiRNA production in gymnosperms and angiosperms including eudicots and monocots was very ancient and conserved, while the transcript regulator miRNAs with rapid diversity were non-conserved or lineage-specific.

### 3.3. The Functional and Expression Diversity of Disease Resistance-Associated MiRNAs between Eudicots and Dicots

Lineage- or species-specific disease resistance-associated miRNAs were continually present or absent during the plant evolution process. However, some miRNAs with similar sequences had obvious functional diversity. For example, miR482/miR2118 in eudicots and gymnosperms mostly targeted *NB-LRR* genes (the bottom panel of Figure 9) and initiated the generation of 21-nt phasiRNAs. However, most of the target transcripts of miR2118 in monocots were noncoding sequences and specifically expressed in the stamen [61,62]. Also, the miR2118 in rice had undergone a special tandem repeat expansion (the middle panel of Figure 9). This phenomenon was also observed in premeiotic and meiotic anthers in maize [70]. The miR2118 initiated the phased siRNA in male reproductive organs in monocots. Therefore, a functional switch occurred in miR482/miR2118 between eudicots and dicots. Additionally, miR482/miR2118 in litchi has also undergone a tandem duplication (the bottom panel of Figure 9) and has evolved lineage-specific functions, such as regulation of zinc-finger proteins, kinase genes, and some noncoding RNAs [69]. In strawberry, miR482/miR2118 was also found to target *non-NB-LRR* genes [71]. This clearly demonstrates the rapid functional diversity of disease resistance-associated miRNAs during the evolution process.

Tae-miR3117b had higher expression levels in the seedling stage of AABB and AABBDD *Triticum* species, while it had lower expression levels in DD species (Figure 2, Supplementary Table S3 and the middle panel of Figure 9). For miR3117a and miR3117b, we only detected one and five reads in GSM803130 (GEO dataset) of sorghum, and none of reads were detected in rice and maize in the GEO small RNA datasets. For miR7757, none of reads were detected in the three species. It may indicate the expression variation in grasses. In addition, the rapid expansion of miR3117 in *Triticum* subgenome A may indicate its rapid evolution on a small timescale. Taken together, the rapid birth/death, functional, and expression level variations were dramatically dynamic in disease resistance-associated miRNAs during selection and the evolutionary pathway in plants.

## 4. Materials and Methods

### 4.1. Identification of Disease Resistance-Associated MiRNAs

To identify the disease resistance-associated miRNAs in wheat, we first downloaded the miRNA sequences from the miRbase (http://www.mirbase.org) [40], PNRD (http://structuralbiology.cau.edu.cn/PNRD) [41], and PmiRExAt (http://pmirexat.nabi.res.in) [42] websites. To detect the miRNA targets, we performed target predictions in wheat with CDS and UTR sequences using the Targetfinder program [43]. Targets with a score less than four were used for further analysis. We selected the miRNAs with target genes containing NB-LRR domains as disease resistance-associated miRNAs. Using the BLAST program, we mapped these miRNAs to the wheat genome and then used the mfold website (http://unafold.rna.albany.edu/) to confirm their precursor sequences.

### 4.2. The Evolution Path of Disease Resistance-Associated MiRNAs

To detect the presence/absence of disease resistance-associated miRNAs in grasses and *Triticum* species, we first downloaded the genome sequences of various grass species including *Oryza sativa* (v7), *Brachypodium distachyon* (v3.1), *Zea mays* (PH207 v1.1), and *Sorghum* (v3.1.1) from the pytozome website (http://www.phytozome.net); *Hordeum vulgare* (IBSC_v2) from the ensemble plant website; and *Triticum* species including *Triticum aestivum* (TGACv1), *Triticum durum* (v1), *Aegilops tauschii* (v1), *Triticum urartu* (v1), *Triticum monococcum* (v1), *Aegilops sharonensis* (v1), and *Aegilops speltoides* (v1) from the URGI website (https://wheat-urgi.versailles.inra.fr/Seq-Repository/Assemblies). Next, we aligned these miRNAs sequences to perfect matches in the genomes of grasses and *Triticum*. According to the presence and absence of these miRNAs, we developed a birth and death phylogenic tree with the evolutionary time scale of these species.

### 4.3. Small RNA Datasets

The small RNA datasets used in this analysis were downloaded from the NCBI GEO database (Supplementary Table S2). The small RNA datasets of diploid, tetraploid, and hexaploid wheat from GSM1364784-GSM1364805 included various developmental stages such as seedling, spike, and seed tissue [72]. The stressed small RNA datasets from 72 libraries, including heat, light and UV, were from SRR3721341-SRR3721412 [49]. The small RNA datasets were responsive to biotic stresses, such as powdery mildew infection, barley yellow striate mosaic *virus*, and *Fusarium* stress from GSM675614-GSM675615 [50], GSM1508254-GSM1508255 [73], and GSM803793-GSM803794, respectively, and abiotic stresses, such as high and low temperatures and heat stress from GSM1294656-GSM1294657 [51] and GSM675616-GSM675617 [50], respectively; salinity and PEG stress from GSM1294656-GSM1294658 and GSM1294656-GSM1294659, respectively [51]; and cold stress from SRS1512057, SRR3690677, SRR3690679, and SRR3690680 [52]. The degradome datasets of SRP076763 [52], GSM911923, and GSM911924 [55] were downloaded from the GEO database.

### 4.4. MiRNA Expression Analysis

MiRNA read normalization as transcripts per million (TPM) was performed for each sample in the small RNA datasets downloaded from the GEO database. The expression levels were shown with the MeV software (http://mev.tm4.org/, version_4.9.0, GitHub, San Francisco, CA, USA).

### 4.5. The Target Gene Analysis

To understand the miRNA targets, we used the Targetfinder program [43] to predict the target genes with a score less than four in the wheat CDS and UTR sequences. To validate whether the miRNAs can indeed cleave the predicted targets, we first downloaded the Degradome datasets from the GEO database, including GSM911923 (cold treatment) and GSM911924 (control treatment) by Tang et al. [55] and SRP076763 for control and cold treatment by Song et al. [52]. Then, we used the four degradome libraries to confirm the target cleavage sites in the wheat CDS and UTR sequences with the CleaveLand program [56]. The cleavage sites were classified as 0, 1, 2, 3, and 4 categories according to the abundance of reads in the cleaved sites along the whole transcripts with a *P*-value less than 0.05. At the cleaved sites, the categories represent as the following: category 4, just one read located at that position; category 3, >1 read but below or equal to the average depth of coverage on the transcript; category 2, >1 read above the average depth but not the maximum on the transcript; category 1, >1 read, equal to the maximum on the transcript when there is >1 position at maximum value; and category 0, >1 read equal to the maximum on the transcript when there is just 1 position at the maximum value.

To identify the domain categories of these targets, we used the Hmmscan program against the Pfam database with an *E*-value less than 10^−10^ to identify NB and LRR domains [53]. We also used the MARCOIL program to identify CC motifs with a threshold probability of 50 [54]. The regulatory network between miRNAs and their targets was shown using the Cytoscape software (http://www.cytoscape.org/, version 3.6.1, U.S. National Institute of General Medical Sciences (NIGMS), USA) [74].

To show the target consensus regions, we used the MEME program (http://meme-suite.org/tools/meme, version 5.0.4, National Institutes of Health, USA) to identify the motifs [75]. To understand target gene clustering, we used the CLUSTALW program in the Bioedit software (http://www.mbio.ncsu.edu/bioedit/bioedit.html, version 7.2.5, Tom Hall, Ibis Therapeutics, A division of Isis Pharmaceuticals, Carlsbad, CA, USA) to align the sequences, removed the high variation sequences manually, and then used the MEGA program (version 10.0.4) with the neighbor-joining method to draw the phylogenetic tree. The parameters such as poisson model, uniform rate among sites, homogeneous pattern, and pairwise deletion for the missing data were selected [64].

### 4.6. The Expression Analysis of Target Genes

To investigate the response of target genes to disease, we downloaded the expression data from the website of http://wheat-expression.com/. Then we extracted the gene expression value of each target from these datasets including the informations of pathogen infections such as *Fusarium graminearum*, *stripe rust*, *Powdery mildew*, *Zymoseptoria tritici*, and flg22. These targets with the TPM (Transcripts Per Million) value more than five and the upregulated fold change more than 2 times after infection by pathogens were identified as upregulated.

### 4.7. Identification of Phased Clusters among the Target Transcripts

According to the small RNA distribution on the target transcripts of disease resistance-associated miRNAs, we identified the phased clusters using the Short Stack package with a phased score of more than 15 [76] and showed the phased distribution of the small RNAs with the IGV package [63].

## 5. Conclusions

MicroRNAs, as an ancient posttranscriptional regulator, have a conserved regulation mechanism in eukaryotic organisms. MiRNAs have evolved independently in the lineages since a lack of universally conservation among plants and animals [77]. In plants, they also evolved lots of lineage-specific miRNAs to be involved in the gene regulation networks. Not like the development-associated miRNAs, for the disease resistance-associated miRNAs, there is no common miRNA between eudicots and monocots; however, the capability of functioning is very similar. Both of them initiate triggering the production of the 21-nt phased siRNAs in their target sites. Moreover, they could respond to various biotic stresses. Therefore, the disease resistance-associated miRNAs with conserved regulatory mechanism on a large or small timescale have a rapid plasticity either in sequences, functions, expression level, or gene copy number in the struggle process against various environments stressed by the pathogens. It would provide the foundation for the molecular study of these miRNAs in wheat, which can be investigated using transgenic wheat lines by strategies of overexpression such as artificial miRNAs and knockdown such as STTM (Short Tandem Target Mimic) or CRISPR (Clustered Regularly Interspaced Short Palindromic Repeat).

## Figures and Tables

**Table 1:** The number of genome loci for disease resistance-associated miRNAs in grasses and *Triticeae* species.

miRNA Name	Seq (5′-3′)	Length (nt)	*Oryza sativa*	*Zea mays*	*Sorghum bicolor*	*Brachypodium*	*Hordeum vulgare (HH)*	*Triticum aestivum (AABBDD)*	*Triticum durum (AABB)*	*Aegilops tauschii (DD)*	*Triticum urartu (AA)*	*Triticum monococcum (A*^m^A^m^)	Aegilops sharonensis (S^sh^S^sh^)	*Aegilops speltoides (S*^s^S^s^)
tae-miR3117a	UGAGAAAGGACUGCAUCAUCU	21	0	0	0	0	0	8	3	2	3	1	3	3
tae-miR3117b	UGAGGAAGGACUGCAUCAUCU	21	4	0	0	5	1	252	23	7	151	5	2	5
tae-miR3084a	UAAUCUUCUGGAUACAUGCUUA	22	0	0	0	0	0	1	1	1	1	0	0	0
tae-miR5071a	UCAAGCAUCAUAUCAUGGACA	21	0	0	0	0	1	6	2	0	1	0	1	1
tae-miR7757a	ACAAAACCUUCAGCUAUCCAUC	22	1	1	3	0	1	3	1	1	1	1	1	1
tae-miR9863a	UGAGAAGGUAGAUCAUAAUAGC	22	0	0	0	3	1	3	1	2	1	2	2	1

**Table 2:** Validated targets with *P*-values less than 0.05 by Degradome data referred from Song et al. [52] and Tang et al. [55]. Number in the parentheses indicate the category of the miRNA-target cleavage.

MiRNAs	Number of Validated MiRNA-Target by Song et al. [52]		Number of Validated MiRNA-Target by Tang et al. [55]		Total Number of Validated MiRNA-Target
	Control	Cold Stress	Control	Cold Stress	
miR3084	1(0)	2(0)	3(0)	3(0)	5
miR5071	5(0); 1(2); 1(4)	7(0); 1(2); 1(4)	11(0); 1(1); 2(2)	11(0); 1(1); 1(2); 1(4)	22
miR7757	1(1)	0	2(0)	1(0)	3
miR9863	9(0); 1(1)	7(0); 1(2)	5(0); 1(1); 1(3)	6(0); 1(3)	16

## Data Availability

The small RNA datasets used in this study were downloaded from the NCBI GEO database (https://www.ncbi.nlm.nih.gov/gds/). The mature miRNAs were downloaded from the miRbase (http://www.mirbase.org), PNRD (http://structuralbiology.cau.edu.cn/PNRD), and PmiRExAt (http://pmirexat.nabi.res.in) websites. The wheat genome (TGACv1) and other *Triticeae* genomes were downloaded from ftp://ftp.ensemblgenomes.org/pub/plants/pre/fasta/triticum_aestivum/ and https://urgi.versailles.inra.fr/download/iwgsc/TGAC_WGS_assemblies_of_other_wheat_species/. The grass genomes were available from ftp://ftp.ensemblgenomes.org/pub/plants/release-37/fasta/ and http://www.phytozome.net.

## Supplementary Materials

Supplementary materials can be found at https://www.mdpi.com/1422-0067/20/13/3128/s1. **Supplementary Table S1.** Mapping information for the disease resistance-associated miRNAs in grasses and *Triticeae* species. **Supplementary Table S2.** The small RNA datasets from the GEO database. **Supplementary Table S3.** The expression levels of disease resistance-associated miRNAs in the small RNA datasets from the GEO database, including diploid, tetraploid, and hexaploid wheat from different tissues. **Supplementary Table S4.** The expression levels of disease resistance-associated miRNAs in the GEO small RNA datasets of biotic stressed including *Powdery mildew*, *BYSMV*, and *Fusarium* and abiotic stressed including CK, heat, UV, light, drought, and salinity stresses. The filled colors in gray, yellow, and blue represent the mock (CK), upregulated, and downregulated expressions, respectively. **Supplementary Table S5.** Information on the disease resistance-associated miRNA targets by Targetfinder program. s-e, start loci to end loci in the genome; score, calculated by Targetfinder program. **Supplementary Table S6.** The target cleavage information from the degradome datasets under control and cold stress treatments by Song et al. [52]. and Tang et al. [55], respectively. **Supplementary Table S7.** Phased siRNA initiation by disease resistance-associated miRNAs. **Supplementary Table S8.** The overlapping targets of disease resistance-associated miRNAs. **Supplementary Table S9.** The response to disease by pathogens for the *NB-LRR* genes targeted by disease resistance-associated miRNAs.

## Abbreviations

NB-LRR	Nucleotide Binding sites and Leucine-Rich Repeat
MiRNA	MicroRNA
SiRNA	small interfering RNAs
PTI	PAMP-Triggered Immunity
ETI	Effector-Triggered Immunity
TIR	terminal Toll/Interleukin-1 Receptor
CC	Coiled-Coil
TIR1	Transport Inhibitor Response 1
Flg22	Flagelin 22
NFYA	Nuclear transcription Factor Y, Alpha
RDR1	RNA-dependent RNA polymerase 1
SCL6	SCARECROW-LIKE6
MET2	METHYLTRANSFERASE 2
PhasiRNAs	phased siRNAs
RDR6	RNA-Dependent RNA polymerase 6
*TuMV*	*Turnip mosaic virus*
*TMV*	*Tobacco Mosaic Virus*
Mla	Mildew resistance locus
TPM	Transcripts Per Million
*BYSMV*	*barley yellow striate mosaic virus*
H	hour
*Pgt*	*Puccinia graminis f. sp. Tritici*
*Pst*	*Puccinia striiformis Westend. f. sp. tritici Eriksson*
